# Machine Learning-Based Behavioral Diagnostic Tools for Depression: Advances, Challenges, and Future Directions

**DOI:** 10.3390/jpm11100957

**Published:** 2021-09-26

**Authors:** Thalia Richter, Barak Fishbain, Gal Richter-Levin, Hadas Okon-Singer

**Affiliations:** 1 Department of Psychology, School of Psychological Sciences, University of Haifa, Haifa 3498838, Israel; galrichterlevin@gmail.com (G.R.-L.); hadasos@gmail.com (H.O.-S.); 2 Faculty of Civil and Environmental Engineering, Technion—Israel Institute of Technology, Haifa 3200003, Israel; fishbain@gmail.com

**Keywords:** machine-learning, behavioral diagnosis, depression, diagnostic tools

## Abstract

The psychiatric diagnostic procedure is currently based on self-reports that are subject to personal biases. Therefore, the diagnostic process would benefit greatly from data-driven tools that can enhance accuracy and specificity. In recent years, many studies have achieved promising results in detecting and diagnosing depression based on machine learning (ML) analysis. Despite these favorable results in depression diagnosis, which are primarily based on ML analysis of neuroimaging data, most patients do not have access to neuroimaging tools. Hence, objective assessment tools are needed that can be easily integrated into the routine psychiatric diagnostic process. One solution is to use behavioral data, which can be easily collected while still maintaining objectivity. The current paper summarizes the main ML-based approaches that use behavioral data in diagnosing depression and other psychiatric disorders. We classified these studies into two main categories: (a) laboratory-based assessments and (b) data mining, the latter of which we further divided into two sub-groups: (i) social media usage and movement sensors data and (ii) demographic and clinical information. The paper discusses the advantages and challenges in this field and suggests future research directions and implementations. The paper’s overarching aim is to serve as a first step in synthetizing existing knowledge about ML-based behavioral diagnosis studies in order to develop interventions and individually tailored treatments in the future.

## 1. Introduction

Depression exerts a major impact on human society, as manifested by its growing prevalence. Worldwide, 264 million individuals of all ages are reported to suffer from depression, making depression one of the most common psychiatric disorders [1]. Indeed, the World Health Organization (WHO, Geneva, Switzerland) ranked depression as the single largest contributor to global disability (7.5% of individuals with disabilities in 2015). Furthermore, depression leads to considerable health and functional losses by impairing the individual’s ability to function at work or school or to cope with daily life [2]. Finally, depression is a significant risk factor for suicide (up to 800,000 cases per year worldwide) [2]. Therefore, depression places a tremendous emotional burden on patients and their families, as well as a financial burden on society.

In the last few decades, medical and clinical psychiatric research has made significant progress in developing treatments and medications for mental disorders, and specifically for depression and anxiety (e.g., the quantum leap caused by the invention of Selective Serotonin Reuptake Inhibitors (SSRIs) [3]. Nevertheless, the recurrent and chronic course of depressive mood disorders (i.e., major depression disorder (MDD) and dysthymia) [2] and the moderate efficacy rates of treatments [4,5] indicate that our understanding of these disorders is limited. Moreover, the high comorbidity rates with other psychiatric disorders [6] have made it clear that some of the difficulties in treating mood disorders result from inaccurate diagnoses. Hence, novel objective assessment tools need to be integrated into the clinical assessment procedure in order to enhance diagnosis objectivity and accuracy. In turn, such tools may lead to individually tailored treatments designed to treat those who do not benefit from common practices.

The procedure for diagnosing psychiatric disorders is currently based on self-reports provided in clinical interviews [7]. Self-reports are subject to various biases [8]. Such self-report biases were also found in questionnaires designed to assess the symptoms of disorders for psychiatric studies [9]. One of the main problems of diagnoses based on self-reports is their highly explicit nature, which may cause individuals to malinger or may lead to unintentional social desirability [10]. In addition, because the diagnosis is based on self-reports and subjective clinician assessments, the same patient may receive multiple and sometimes contradictory diagnoses during the course of treatment [11].

Although self-report questionnaires and structured interviews typically exhibit excellent validity and reliability when performed by trained clinicians [12,13], they may sometimes lack the psychometric properties of sensitivity and specificity, which are valuable in differentiating one disorder from another or healthy from non-healthy individuals. For example, correlations between scales that measure anxiety and those that measure depression range between 0.5–0.7 [14,15]. Therefore, self-report-based diagnosis is limited in its ability to sensitively pinpoint unique characteristics of each mental disorder and differentiate between disorders [7].

Finally, some populations may encounter difficulty in reporting their symptoms, such as children, individuals with developmental or communication disorders, individuals with organic or neurological deficits (such as mental retardation or dementia), or even people with low mental health literacy e.g., [16]. The ability to collect reliable self-reported information from these populations is limited.

The evidence outlined above suggests that the psychiatric diagnostic process would benefit greatly from adding data-driven tools that can enhance diagnostic accuracy and specificity alongside the clinical interview and self-report questionnaires. Behavioral and neurophysiological information may meet this need, as such information facilitates both scientific and clinical analyses of objective data that are not exclusively based on explicit overt symptoms. Such tools are beginning to gain popularity, among other reasons as a result of technological developments. For example, the Test of Variables of Attention (TOVA) is a behavioral objective assessment tool that has exhibited adequate results and has already been in use for years in diagnosing attention deficit hyperactivity disorder (ADHD) [17].

In addition to objective assessment tools, such as the TOVA or MRI scanners, which make it possible to collect objective data, advanced data analysis frameworks also have the potential to aid in diagnosis. Newly developed advanced data techniques have motivated researchers in the fields of psychiatry and computer science to combine forces and seek new methods of analysis, beyond classical statistical approaches. These efforts have yielded fruitful collaborations in clinical studies based on Machine Learning (ML) [18].

## 2. Machine Learning Analysis in Psychiatry

The aim of ML analysis is to uncover general principles underlying a series of observations without explicit instructions being provided. ML methods differ from classical statistical approaches in that they are data-driven and rely on as few formal assumptions as possible. Furthermore, they can generate structured knowledge from large-scale data [18]. Finally, these methods allow detection of complex, non-linear, and high-dimensional interactions that may inform predictions, even in the presence of major instrumental and scoring noise [19]. Among the most prevalent of these analysis techniques are the following: support vector machines—a supervised model that makes predictions by identifying observations in the data that are typical of the categories to be differentiated; modern neural-network algorithms—a supervised model that makes predictions based on a nonlinear, multilayer variant of linear regression; and cross-validation procedures—a two-step procedure used as the standard for estimating the ability of a learning model pattern to be replicated in future data samples by training the algorithm on the majority of the data and then testing it on the omitted sample, and then repeating this procedure with different random splits of the data. These methods are now widely used in clinical studies, each for a different purpose (see [18] for elaboration on ML techniques in psychiatry).

In recent years, clinical studies employing ML analysis in psychiatry have achieved promising results in four major domains: (i) detection and diagnosis of mental disorders; (ii) disease prognosis, treatment, and patient support—a field focusing mainly on the use of ML to predict long-term prognosis outcomes prior to or after diagnosis; (iii) public health applications, which include assessments of mental health in various populations, tracking mental health following a dramatic event or disaster, and creating risk models to improve health system services; and (iv) research and clinical administration focusing on improving resource allocation methods and research methodologies [20].

The current paper focuses on ML analysis approaches for the detection and diagnosis of depression disorders. It discusses the advances and challenges in diagnosing depression with the aid of ML by reviewing two common types of studies: (i) studies that entail training a dataset of prior diagnoses in order to predict diagnosis and (ii) studies that use ML analysis to differentiate between psychiatric disorders with similar symptomatology. Both types of studies have focused primarily on neuroimaging data, particularly MRI/fMRI, EEG and PET [20].

A growing number of ML-based studies have shown promising results using neuroimaging data for diagnosing depression disorders. For example, Rubin-Falcone et al. [21] differentiated between bipolar disorder and MDD by measuring grey matter volume and analyzing the data using ML techniques. Sato et al. [22] applied ML analysis to resting-state fMRI data from children and adolescents in order to classify brain networks as typical or atypical. The children that the algorithm identified as having atypical networks scored higher on questionnaires of externalizing behaviors (i.e., rule-breaking behaviors and aggression) and internalizing behaviors (i.e., anxiety/depression, withdrawal/depression, and somatic symptoms). Both of these types are known to be associated with development of psychopathology in adulthood. Rosa et al. [23] successfully classified participants into those with MDD diagnoses or healthy control participants without no psychiatric diagnosis. The classification was based on patterns of brain connectivity revealed by two fMRI datasets measured, while participants performed cognitive tasks. For extensive reviews of ML-based neuroimaging studies for diagnosis of depression, see Patel et al. and Gao et al. [24,25].

These studies have exhibited favorable results. Yet, despite their scientific importance, the potential of these methods to serve as ordinary diagnostic tools is limited because neuroimaging measurement is costly and not commonly available. Hence, objective assessment tools are needed that can be more easily integrated into the regular psychiatric diagnostic process in terms of cost and mobility. In contrast to neuroimaging techniques, behavioral assessment data are easily collected while still maintaining objectivity. Hence, behavioral assessment is an excellent candidate to serve as an ML-based diagnostic support system. The current paper is a descriptive narrative review of ML-based studies that focus on behavioral data in diagnosing depression. Due to the novelty of the field, most of these studies are relatively recent, and to our knowledge, this is the first attempt to unify and organize them in one overview. The current paper seeks to summarize the main developments, challenges, and trends in the field. Therefore, wherever possible, we refer the reader to comprehensive reviews with a broad scope.

Method used in literature search: Search platforms: We used Google Scholar, Scopus and PubMed, which are believed to be comprehensive and to incorporate all relevant knowledge fields.

Search keywords: [machine learning] AND [depression] AND [diagnosis] Or- [machine learning] AND [depression] AND [behavioral diagnosis].

Inclusion criteria: English-language publications published in the past eight years in peer-reviewed journals or conferences.

To ensure the inclusion of major trends and developments only, each time highly similar studies were detected, only the first (prioritized by relevance) was chosen. Furthermore, we followed comprehensive reviews in the field in order to ensure both novelty and relevance.

## 3. Machine Learning Analysis Based on Behavioral Assessment in Psychiatry

Behavioral data are collected in various creative ways that can be categorized into two main groups: (a) laboratory-based assessments, in which a special paradigm is required in order to obtain the information, and (b) data mining, in which the data are retrieved from existing sources. The latter group can be divided into two sub groups: (i) social media usage and movement sensors data and (ii) demographic and clinical information. Naturally, there is some overlap between the categories.

## 4. Laboratory-Based Assessments

Laboratory-based assessments are paradigms that involve affective sensing. Disturbances in the expression of affect reflect changes in mood and also interpersonal style. Hence, these expressions can serve as a key index of a current depressive episode [26]. Affective sensing, i.e., the sensing of affective states, is one of the novel and emerging uses of information technology. Automatic face tracking in videos, measurements of facial activity, recognition of facial expressions, analysis of affective speech characteristics, physiological effects, and other non-verbal cues can all be used as vicarious measurements of changes in affective state that may inform diagnosis [26]. Several ML-based studies have applied these methods in predicting pathological symptoms or psychiatric disorders. For example, children with and without internalizing disorders performed a 90-s mood induction behavioral task designed to elicit motor reactions in three different stages: potential threat, startle, and response modulation. These stages were achieved by gradually exposing the participants to realistic-looking rubber snakes. Wearable sensors recorded motion, which was then analyzed by an ML-based algorithm that predicted participant classification to a disorder or a control group [27]. The algorithm exhibited 80% diagnostic accuracy. Similarly, after children performed a 3-min mood induction speech task, in which they were instructed to prepare and give a three-minute speech and told they would be judged based on how interesting their speech is, ML-based analysis of audio data was used to identify those with internalizing disorders that may be predictive of depression by identifying differences in pitch altitude, speech inflections, and other affective speech-related components [28]. The algorithm reached 80% diagnostic accuracy. Children with internalizing disorders were generally found to exhibit low-pitched voices with repetitive speech inflections and high-pitched responses to surprising stimuli. These results point to the potential use of audio and motor data in detecting children prone to anxiety and depression. Early detection may facilitate implementation of effective intervention and prevention programs.

In another study, the researchers examined videos of adult participants in order to predict severity of depression symptoms based on the videos. Specifically, data were recorded from videos of participants’ faces in order to obtain spatial and temporal information regarding appearance, static expressions, motion across frames, and facial dynamics such as expressions and micro-expressions. The videos included participants reading sections from a book or answering questions such as “what is your favorite dish” or “discuss a sad childhood memory”. The algorithm detected patterns of facial expressions and dynamics, which successfully predicted the severity of depression symptoms [29]. The researchers compared their results to other ML-based studies that used both audio and visual videos and found that their study, although based on visual videos only, had equal or higher accuracy rates (see similar studies conducted by Kang et al. and Maridaki et al. [30,31]). In contrast, Joshi et al. [26] recorded both visual and audio information from videos of depressed and non-depressed participants in order to predict diagnosis based on ML classification. Recordings included participants watching movie clips, watching and rating affective pictures, reading sentences containing affective content, and an interview between participants and a research assistant. Intra-facial muscle movements and head and shoulder movements were measured by computing spatiotemporal interest points, together with various audio features, such as fundamental frequency, loudness, and intensity. When diagnoses were predicted based solely on audio or solely on visual information, accuracy rates were 81–83%. When prediction was based on both types of information, accuracy increased to 91% (see a similar study by Victor et al. [32]). Smith et al. [33] examined whether vocal information will contribute to ML-based prediction of depression among elderly adults as well, as this population (age 65 or above) is typically excluded from clinical studies. Participants were recorded while giving free speech and reading aloud from a book. Prevalent audio features were recorded and analyzed. Prediction accuracy ranged between 86%–92%.

In another study, researchers aimed to predict depression and anxiety based on natural gait patterns. A digital camera recorded participants walking, and the position and temporal dynamics of 18 key body points were measured. The ML algorithm prediction accuracy was 86% for depression and 78% for anxiety. Depressed and anxious individuals were found to have greater walking-movement variance [34].

In addition to these studies based on bodily expressions, several attempts have been made to detect physiological biomarkers that may inform affect and predict diagnosis. Proteomics (i.e., the study of proteins) allows for unbiased data-driven detection of novel protein biomarkers related to functional abnormalities involved in MDD pathophysiology [35]. In addition, reduction in heart rate variability (HRV) is associated with depression and anxiety disorders and with severity of depression symptoms [36]. Kim et al. [37] focused on a combination of proteomics and heart rate dynamics. Heart activity at rest was measured by electrocardiogram (ECG), and quantitative serum proteome profiles were analyzed using pooled serum samples. Participants were MDD patients or healthy controls. None of the participants were taking psychiatric medications and none had been diagnosed with heart disease or endocrine or immune abnormalities. Basal measurements were taken. Participants fasted and refrained from smoking and caffeine for two hours prior to testing, and from alcohol for 12 h before ECG recording. Testing times were from 9:30 to 11:30 a.m. Participants were instructed to minimize their movements and breathe regularly with their eyes closed while in a recumbent position. Candidate proteins were quantified using multiple reaction monitoring (MRM), a specific and sensitive mass spectrometry technique that can selectively quantify compounds within complex mixtures. An ML algorithm was applied to select proteins and HRV parameters that can help in classifying MDD patients and healthy controls (i.e., feature selection). The algorithm predicted diagnosis with 80% accuracy rates.

In addition to these studies, which are based on rather small-scale samples, several studies have used extensive health examination surveys that tested participants’ blood and urine in order to analyze large numbers of possible biomarkers to predict depression. For example, in Dipnall et al. [38], 21 biomarkers were found to be predictive. The top three were red cell distribution width, serum glucose, and total bilirubin. In Sharma and Verbeke [39], 28 biomarkers were found to be predictive. Those that made the greatest contribution were platelets, triglycerides, alkaline phosphatase, creatinine 24-h urine, and neutrophil granulocytes. Important demographic information factors, such as gender, age, race, smoking, food security, Poverty Income Ratio, Body Mass Index, physical activity, alcohol use, medical conditions, and medications, were inserted into the model as covariates. Biomarkers studies have been used to differentiate between psychiatric populations as well. For example, Wollenhaupt-Aguiar et al. [40] and Tomasik et al. [41] analyzed blood biomarkers to differentiate between unipolar and bipolar depression. Participants were patients with unipolar or bipolar depression or healthy controls. Exclusion criteria included past diagnosis of heart disease or endocrine or immune abnormalities. In the Tomasik et al. [41] study, participants were asked to collect five separate samples of dried blood taken after at least six hours of fasting and to submit these tests by mail. These biomarker studies effectively utilize common and accessible procedures (blood and urine tests) to add objectivity and specificity to the diagnostic procedure. Although heart rate measurements and blood and urine tests require special equipment, they are still cheaper and more accessible than neuroimaging machines. Additionally, if ML methods become implemented on a regular basis, the laboratory tests required for analyzing the data can be better integrated in future health care at lower costs.

Several studies focused on paradigms that measure cognitive-behavioral performance. Cognitive-behavioral paradigms are time-consuming and data collection currently requires a great deal of effort. Nevertheless, these paradigms can provide highly objective information that can shed light on the unique deficits of each patient.

Cognitive biases, which are defined as enhanced or preferential processing of stimuli that have an emotional valence or meaning relevant to the disorder [42], are frequently presented by depressed and anxious individuals e.g., [43,44,45]. These biases maintain disordered affect and play a key role (either causal or contributory) in the onset and maintenance of these conditions as well as in the possibility of recovery [45]. Therefore, they were chosen as the focus of studies aiming to develop an ML-based diagnostic support system to classify different disorders. For example, participants with clinical and sub-clinical levels of anxiety and/or depression performed a test battery of cognitive behavioral tasks, each examining a different type of bias [46,47]. The battery included six prevalent cognitive tasks, with modifications allowing it to test both automatic and non-automatic reactions to emotional stimuli. For example, the expectancy bias task measured overt decisions regarding whether a negative or positive described situation will happen sometimes in the future, while the decision reaction time was used in order to examine the automatic inclination. By means of detecting a performance pattern unique to each disorder, an ML algorithm predicted diagnosis with up to 80% accuracy. These findings, which are based on cognitive mechanisms rather than on self-reports of behavioral or emotional symptoms, may enable clinicians to achieve increased diagnostic precision. Furthermore, this tool may increase the confidence of both clinicians and patients in the diagnosis by equipping them with an objective assessment tool.

Analyses of cognitive behavioral performance have yielded highly objective information that can shed light on the unique deficits of each patient, even though this type of data requires relatively special effort to obtain. A core feature of this approach is its reliance upon cognitive biases, which have been found to be core characteristics of psychiatric disorders. Therefore, this type of analysis can make a substantial contribution to diagnosis accuracy, as well as provide a unique profile that can serve as the basis for future individually tailored treatments.

Overall, affective sensing serves as a valuable tool for measuring verbal and non-verbal cues that can be used as a vicarious measurement of affective state changes. These cues may inform diagnosis without the need to rely upon explicit self-reports. If integrated in the diagnostic procedure, they can be valuable in choosing the most accurate diagnosis and in making a differential decision.

Nevertheless, laboratory-based assessments have a low probability of assessing risk for depression without any prior indications. Since these assessments require special effort, individuals who undergo them are likely to participate for an already-known help-seeking reason. One way to overcome this problem is to deploy these cognitive/behavioral assessments at routine annual checkups at which “red flags” may arise even if there was no prior indication.

ML-based affective sensing has been utilized in other psychiatric disorders as well, in order to differentiate between disorders that share some common aspects. Affective features and concept-level text analyses have been applied in diagnosing autistic spectrum disorder and schizophrenia—disorders marked by a range of communicative and linguistic difficulties. Data were obtained by asking participants to reply to six open questions, four of which concerned the patients and their families, the person closest to the patient, and the patients’ interests and childhood. Two of the questions were more abstract and asked why people get sick and why people believe in God. The results were more accurate for detecting schizophrenia than for detecting autism, with the highest in the 80% range for schizophrenia and the 63% range for autism. The researchers hypothesized that this finding was due to the fact that some symptoms are shared by both disorders, namely symptoms of negative thought disorder (e.g., poverty of speech or poverty of speech content), while patients with autism typically do not present the positive symptoms (e.g., pressure of speech or derailment) that schizophrenia patients often present [48]. Demetriou et al. [49] used cognitive-behavioral methods for diagnosing different psychiatric disorders that share a common social difficulty, namely, autistic spectrum disorder, early psychosis, and social anxiety disorder. The researchers analyzed patients’ performance on several tests of cognitive-behavioral functions. Sixteen features of these tests successfully differentiated between the groups with up to 90% accuracy. The control group, which had no psychiatric diagnosis, was differentiated from the clinical groups by social cognition and visuospatial memory functions. In addition, a distinct profile cluster drawn from social cognition, visual learning, executive functions, and mood distinguished the groups with neurodevelopmental diagnoses (early psychosis and autistic spectrum disorder) from the social anxiety group. These studies may inspire future research aimed at differential diagnosis of depression to develop paradigms targeting the unique cues or mechanisms of depression rather than its shared characteristics with other disorders, in order to achieve better differentiation.

## 5. Data Mining

### 5.1. Social Media Usage and Movement Sensors Data

Social media data provide helpful indicators about states of physical and mental health. Computational social scientists are interested in research on depression since depression may influence a range of behaviors and patterns of communication that can be reflected on social media [50] (for an extensive review of ML-based and classical statistics-based diagnosis of depression and other psychiatric disorders from social media data, please see Guntuku et al. [51] and Hassan et al. [52]). Studies that analyze social media usage typically obtain data from either Facebook or Twitter by analyzing qualitative or quantitative characteristics of the content of posts or of patterns of use [51].

ML algorithms have detected several abnormal patterns of social media usage among depressed individuals. For example, through an analysis of Twitter data, De Choudhury et al. [53] found a reduction in social activity, elevated negative affect, highly clustered ego networks (in which social interactions are depicted from the ego’s point of view), heightened relational and medicinal concerns, and more expressions of religious involvement among depressed individuals. The analysis collected features such as engagement (e.g., number of posts per day), affect, linguistic style, and depression language. Classification accuracy was around 71%. Furthermore, in a study that analyzed predictive features measuring affect, linguistic style, and context from participants’ tweets, the ML algorithm was able to predict depression with 85–95% accuracy rates, even when the analysis was restricted to content posted before the first depression diagnosis. The feature that contributed most to the analysis was an increase in the use of negative words among the depressed group, and the second highest contributing feature was a decrease in positive language in the depressed group [50].

Reece and Danforth [54] also detected depression with 70% accuracy from Instagram photos posted prior to diagnosis by analyzing color and metadata components and using algorithmic face detection of Instagram photos. Some of the analyzed features were related to content (e.g., human figures, setting, and time of day), statistical properties of images at a per-pixel level, such as average color and brightness values, Instagram metadata, such as number of “likes”, and platform activity measures, such as usage and posting frequency. The results indicated that photos posted by depressed participants tended to be bluer, darker, and grayer. Furthermore, the posts of depressed participants attracted more comments and fewer likes than those of healthy controls, and higher posting frequency was associated with depression. Depressed participants were more likely to post photos with faces, but had a lower average face count per photograph than healthy participants. Finally, depressed participants were less likely to apply Instagram filters to their posted photos.

These results suggest that the onset of depression may be detectable from social media data several months prior to diagnosis. A similar study, however, found that tweets from the most recent 6–16 weeks were more accurate in recognizing depression than those from more distant periods of measurements, with an accuracy rate of 69%. Depressed participants were found to have a higher ratio of negative words and more frequent tweets and retweets [55]. Chiong et al. [56] aimed to combine several social media platforms and examine whether ML algorithm will predict depression, even when the posted text does not explicitly contain specific keywords such as ‘depression’ or ‘diagnosis’. The researchers tested linguistic features of two public Twitter datasets to train and test the ML models, and then another three non-Twitter depression-diagnosis-only datasets (sourced from Facebook, Reddit, and an electronic diary) to test the performance of the trained models against other social media sources. Predication accuracy rates ranged between 82–92%.

In another study that analyzed mobile phone usage and predicted depression with 76–81% accuracy rates, depressed participants were found to have fewer saved contacts on their devices, spend more time on their mobile devices, and make and receive fewer and shorter calls and send more text messages than healthy controls [57]. Finally, an ML-based study analyzed a set of attributes, namely emotional processes, temporal processes, and linguistic style, among Facebook posts to predict depression diagnosis. The study identified several attributes that contribute to depression diagnosis and are highly linked to its symptoms and etiology, among them strong feelings of sadness for long periods of time (weeks, months, or even years) and in some cases with no apparent reason; engaging in themes of physical and emotional changes associated with puberty, bereavement, starting a family, and retirement; differences between content published during the day versus at night, with daytime content related to feelings of loneliness, stress, and lack of energy; reporting problems related to interactions between family members; and physical illness. Accuracy rates ranged from 60–80% [58]. These findings point to the possibility of collecting important diagnostic data that are not based on self-reports but can still inform clinicians about individuals’ feelings.

In another study, Hou et al. [59] analyzed reading habits in addition to social data usage in order to predict depressive tendencies among students. The researchers measured three dimensions: reading times, reading frequency, and reading span (at most five types of books and periodicals). Diagnostic accuracy was 82%. Results indicated that the higher the risk for depression, the more time students spent reading technical and psychological books and the less time they spent reading novels and amusing books. This study suggests a strong correlation between reading habits and mental health and may contribute to developing prevention programs for students by implementing the algorithm in academic library systems.

Taken together, these studies demonstrate that analysis of social media usage may alert patients and clinicians of the existence of depression even prior to first diagnosis, based on features related to content, visual elements, and patterns of activity. Since social data provide a highly accessible and relatively effortless assessment of depressive behaviors and patterns of communication that are reflected online, in the future, they can assist in diagnosis as well as be used in individually tailored treatments.

Similar to social media usage, information regarding an individual’s daily movements and locations may also inform the prediction of depression, as depression symptoms typically affect patterns of movement and physical activity [60]. Equipping smartphones and wearables to capture moment-by-moment datasets with sensing apps has made it possible to collect datasets passively and in naturalistic settings. Inherent in these datasets are behavioral patterns: routines, rhythms, activities, and interactions that are useful indicators of depression [61].

For example, in an ML-based study that sought to detect depression among older adults living alone, a wrist-worn Actiwatch was used to record physical activity, sleep efficiency, and ambient light exposure every 30 s for a period of 14 days. The prediction accuracy was 70%. The depression group exhibited a significantly low level of daytime activity and higher levels of ambient light exposure. Furthermore, sleep efficiency, defined by measurements of nighttime activity and quality of sleep, was significantly lower among the depression group. [62]. In a similar study, a wristband biosensor device was used to measure step count, energy expenditure, body movement, sleep time, heart rate, skin temperature, and UV light exposure during the daily activities of out-patients with depression. Healthy controls had significantly greater step counts and energy use during the hours from 11:00 am to 6:00 pm. In addition, among patients, sleep time was particularly long during the nighttime hours of 9:00 pm–12:00 am. Moreover, the depressed patients exhibited higher levels of physical calmness during nighttime hours. The prediction accuracy rate in this study was 76% [63].

These studies offer momentary assessments that are more ecological while at the same time maintaining accessibility and objectivity, which do not require laboratory settings.

### 5.2. Demographic and Clinical Information

In recent years, several studies have suggested using accessible and relatively objective demographic and clinical information about patients that already exists in healthcare systems’ databases or that can be easily retrieved from patients without risking self-report biases of clinical symptoms. The use of a combination of ML methods makes it possible to detect specific clinical and demographic factors that predict risk for depression and is time- and cost-efficient. Some of these studies combine sociodemographic factors with physiological factors obtained from medical tests, which are also compatible with the field of biomarker diagnosis.

Sau and Bhakta [64] were able to predict anxiety and depression symptoms by analyzing a list of sociodemographic factors, such as age, academic qualifications, monthly income, employment status, BMI, marital status, presence of hypertension, diabetes or ischemic heart disease, and job profile. Similarly, Oh et al. [65] analyzed large-scale surveys (thousands of participants) that consisted of demographic data (e.g., age and gender), dietary data (e.g., number of meals per day), medical examinations (e.g., currently being treated for asthma), various laboratory tests (e.g., percentage of glycohemoglobin), and other questionnaire data that do not directly assess depression or symptoms (e.g., duration of unemployment and smoking habits) in order to predict depression diagnosis. Diagnostic accuracy was 82%, with each of the factors contributing significantly to the prediction. One interesting finding was that out of 157 rather objective features, the most predictive feature was the individual’s subjective feeling of health. See similar studies by Souza Filho et al. and Nemesure et al. [66,67].

Sau and Bhakta [68] and Su et al. [69] analyzed sociodemographic and medical information of older adults in order to predict anxiety and depression diagnosis and to identify unique high-risk factors specific to geriatric patients. Activities of daily living, self-rated health, marital status, arthritis, and number of cohabitants were found to be the most important predictors of depression in older adults [69]. In another study, the researchers aimed to predict persistent depression among older adults, as more persistent depression symptoms are prevalent among the elderly population. Levels of depression were recorded three times during a period of 12 months. The ML algorithm analyzed demographic information such as age and sex, current medication and physical health conditions and other questionnaire data. The algorithm predicted with 72% accuracy the presence of depressive symptoms after one year of the study, with the ability to function in daily activities as one of the most predictive features [70].

Hochman et al. [71] and Zhang et al. [72] predicted postpartum depression (PPD) among women by analyzing demographics, medication prescriptions, procedures, laboratory measurements, and social determinants of health (in [72]), including characteristics of the built environment, such as distance to public transportation and green space. In Hochman et al. [71], past depression and differing patterns of blood tests were most predictive of PPD. Zhang et al. [72] found specific pregnancy characteristics that were predictive of PPD, such as caesarean section, single motherhood, and mental and physical difficulties during pregnancy. Furthermore, Zhang et al. [72] collected and analyzed information at several time points during pregnancy and updated the prediction accordingly. They suggested that using ML-based diagnosis throughout pregnancy may give care providers the opportunity to take timely actions according to the risk of developing PPD as evaluated by the algorithm, which is constantly updated in response to new information accumulated at repeated visits during the pregnancy.

Instead of collecting data for clinical research purposes, several studies pinpointed patients’ electronic health records (EHR) as an efficient means of obtaining sociodemographic and clinical characteristics, as EHR are routinely collected during treatment and already exist in mental health institutions. Wang et al. [73] applied ML algorithms to the EHR of pregnant women in order to predict PPD. Race, obesity, current anxiety and depression diagnosis, different types of pain, antidepressants, and anti-inflammatory drugs during pregnancy were among the significant predictors, with accuracy rates of 79%. In another study [74], EHRs were analyzed to predict risk of readmission for suicide attempts and self-harm among adult women with serious mental illness (i.e., depression, bipolar disorder, and chronic psychosis). The prediction accuracy was 84%. Predictors included medical comorbidity, history of pregnancy-related mental illness, age, and history of suicide-related behavior. The patients found to be at the highest risk for readmission were women with antecedent medical illness and a history of pregnancy-related mental illness, as were women below age 55 without antecedent medical illness. These results add an important aspect to diagnostic research: in contrast to most of the studies that focus on who may suffer from mental illness, the current results diagnose the level of self-harm risk among already-diagnosed patients, thus allowing for targeted and more specific future interventions.

Zhou et al. [75] suggested that focusing only on EHR may result in a loss of valuable information, since structured EHR data, which are mostly collected for billing purposes, do not include much of the depression-related information that is documented in clinical notes, and specifically in discharge summaries. These researchers applied natural language processing systems and ML classification algorithms to the discharge summaries and EHR of random hospitalized patients with two purposes in mind: identifying depression diagnoses from free-text notes and utilizing unstructured data from clinical narratives to identify patients at high risk for hospital readmission. They found a high prevalence of depression among patients hospitalized with ischemic heart disease. The majority of patients were detected by the EHR analysis, while some were detected only by the discharge summaries analysis.

Demographic and clinical information contains certain characteristics of individuals with depressive symptoms that may be manifested in their personal details. These data already exist, usually in electronic form, and therefore, they offer efficient diagnosis in terms of time and costs. Moreover, they can detect specific clinical and demographic factors that predict the risk for depression as well as the level of dangerous symptoms among already-diagnosed patients. Hence, these data may be used to target specific populations for future interventions.

In conclusion, data-mining methods are accessible and efficient tools that can be integrated in the diagnostic procedure to support diagnosis. In contrast to laboratory-based assessments, they can be excellent ways to notify people about a possible risk for depression by implementing them in public health care systems or by outreach to the public, for example by disseminating triggering ads offering to scan people’s social media and to notify them if they need to seek help.

Although these studies accurately assess the risk for depression, they are limited by the fact that the majority do not include other clinical diagnosis groups besides the depression or the control group for comparison purposes. Hence, their ability to support a differential diagnosis is yet unknown. Future research is advised to examine this issue.

## 6. Advances and Future Implementations of ML-Based Behavioral Diagnosis

As noted throughout this paper, behavioral ML-based methods offer objective and accessible diagnostic measurements in the field of psychiatry. Implementing these methods in psychiatric evaluations alongside clinical interviews and self-report questionnaires may contribute to the specificity and sensitivity of diagnostic decisions, especially in the case of two diagnoses with highly similar symptoms (as in the case of depression and anxiety) or when the population in question is limited in its ability to accurately report their difficulties. More sensitive and specific diagnoses may assist clinicians to select the most successful treatment type, which in turn will lead to better resource allocation and the development of prevention interventions. Machine-based assessments will never be able to replace the integrative perspective of trained clinicians, yet they may improve clinicians’ confidence and help them base their diagnosis on broader and more objective sets of data that include both overt symptoms and more covert cognitive biases. This is also true for self-reports questionnaires, which for the most part have reliability and validity rates similar to the accuracy rates of ML-based diagnosis. Combining the two methods may help increase diagnosis sensitivity and specificity by allowing subtle differences between disorders or between healthy and non-healthy individuals to emerge from in the data, thus making differential diagnosis easier.

Another important advantage of ML-based behavioral diagnosis is its ability to collect highly specific and personal information about individuals, thus contributing to personalized medicine, both for diagnostic purposes and for future implementation in treatments and interventions.

The different behavioral data categories discussed above suggest that extremely personal information can be retrieved from each patient and used to draw both group and individual conclusions. A great deal of research in recent years has shown that personalized medicine and individually tailored treatments improve outcomes in both physical and mental health e.g., [76,77,78]. Therefore, incorporating ML approaches in the diagnostic procedure may greatly improve the efficacy of treatments that rely on its findings. For instance, knowledge derived from cognitive-behavioral performance may be highly beneficial if implemented in cognitive training, such as cognitive bias modification (CBM). CBM seeks to modify cognitive processes to become more adaptive to daily life and has been found to improve psychopathological symptoms [79]. The efficacy of CBM was found to be affected by the intervention selection—a central approach in personalized medicine. Intervention selection targets optimization of intervention efficacy by identifying the most favorable type of intervention for a given individual [80]. ML approaches allow for the selection of those characteristics that contribute most to treatment, without relying on a specific theory. Therefore, these approaches may be highly suitable for such identification. Indeed, when variables previously found by an ML algorithm to increase treatment efficacy were implemented in the course of treatment, treatment outcomes improved [81].

Future research may benefit from combining several methods of behavioral data collection and ML-based analysis in order to obtain as much information as possible that is not based on self-reports and that can be easily retrieved. The goal is to achieve a high degree of accuracy. Based on the findings, protocols of diagnostic procedures may entail obtaining information from several sources, depending on health care resources, for example by combining clinical assessment, social data usage information, EHR information, and neuroimaging, if possible. Moore et al. [82] suggested a similar notion in the form of proof-of-concept evidence for a novel brain assessment approach that includes integration across multiple brain imaging modalities and cognitive tasks that reliably modulate the engagement of the brain systems of interest.

## 7. Current Challenges of ML-Based Behavioral Diagnosis

Despite these remarkable advances and suggested opportunities, ML-based behavioral diagnosis research still entails some substantial difficulties. Lack of consistency in technique accuracy and in the types of algorithms and datasets used is a major issue. More research is needed to combine findings and develop standard techniques that can be embraced by mental health clinicians and institutions. In a meta-analysis, Zulfiker et al. [83] applied the same specific ML-algorithm to datasets from 30 studies of ML-based depression diagnosis. The algorithm achieved higher diagnosis accuracy for each dataset than the original accuracy rates. Similarly, Shani et al. [84] recently developed a protocol for cognitive training analysis, highlighting the need for standardized techniques in order to achieve greater efficacy. In addition to higher accuracy, the development of standardized models may lead to greater collaboration among researchers, data science experts, and clinicians. Such interdisciplinary collaboration is needed to achieve the highest degree of efficacy possible and to make these tools readily accessible for clinical use.

Currently, the majority of ML research is conducted in lab settings, mostly after a real-world diagnosis is given. In order to test clinical utility, more real-time and ecological research settings are needed. For example, studies should be conducted during the actual diagnosis of newly admitted patients to compare clinicians’ diagnoses to ML classifications. Subsequently, patients should be monitored to examine the results of treatment according to a given diagnosis.

Another inherent limitation of ML-based behavioral diagnosis lies in the fact that the classification algorithms are validated against questionnaires/clinical diagnoses, which as mentioned are prone to self-report biases and are not 100% accurate. Related to this point, gold standards are yet to be developed in the diagnostic process of depression disorders, similarly to other psychiatric disorders. For example, Kathol and colleagues [85] found that the diagnosis of depression in cancer patients differed as a function of the diagnostic tool that was used. Angst and Merikangas [86] highlighted differences between diagnosis based on a continuum of symptoms’ severity versus a binary diagnosis of a discrete category. As the diagnosis of depression disorders depends on clinical interviews and questionnaires, which vary depending on various factors, the development of gold standards for diagnosis will enable comparison of new methods, such as ML-based diagnosis, in a more reliable manner. Currently, the degree of objectivity of the analysis, even when fully data-driven algorithms are applied, is limited. Yet, as more such studies are conducted and synthesized together, this bias will be mitigated.

The high cost of studies involving human participants results in small sample sizes in the majority of ML-based psychiatric studies. This may lead to overfitting of the ML model to a specific dataset. Indeed, such bias was found in reviews showing higher accuracies in studies with smaller sample sizes [87,88]. However, this bias was shown to be mitigated when using nested K-fold cross-validation and train/test split approaches [89]. These approaches suggest resampling processes that are designated to test the validity of the ML model in an unseen dataset. The limitation of small samples is gaining more acknowledgment and more studies apply these procedures to control for sample size. As mentioned, greater standardization of sample size control will lead to more quality results.

Finally, in spite of impressive accuracy rates, the results of ML models may still be treated as “black boxes”, mapping given input to classification output while offering little to no explanation for why, for example, specific features are chosen over others during training or how correlations in the training data are represented in feature selection [90]. Interpretability is ML’s counterpart to the human thought process of justifying prediction through a series of logically consistent and understandable choices. Interpretability is critical to integrating ML-based tools in the clinical diagnostic process in order for clinicians to trust the algorithm’s prediction. Chakraborty et al. [90] suggested that in order for ML models to be interpretable, they must integrate several dimensions of transparency and functionality. These researchers reviewed several studies that focused on developing such interpretable models. Future studies may benefit from collaborations between researchers in the fields of computer and human science that will consider the interpretability of the model under development and combine their fields of expertise in order to achieve it.

## 8. Summary

The current paper sought to summarize important research advances in the development of objective behavioral ML-based tools to assess depression. This research field has emerged in response to the need for more objective and individually tailored diagnostic tools for psychiatric disorders that will also be more accessible and routine than neuroimaging machines. The added value of ML analyses combined with these novel tools is tremendous and includes objective data-driven algorithms that can generate structured knowledge from large-scale data and detect complex non-linear high-dimensional interactions.

As discussed, in order to make this knowledge available for clinical use, the various research lines need to be integrated. Despite the many studies examining the use of ML, very few have attempted to integrate the different aspects of the field. The current paper represents a first step in this direction by integrating the different paradigms, reviewing current challenges and making suggestions for future progress.
